# To unite or divide: mitochondrial dynamics in the murine outer retina that preceded age related photoreceptor loss

**DOI:** 10.18632/oncotarget.5614

**Published:** 2015-09-10

**Authors:** Jaimie Hoh Kam, Glen Jeffery

**Affiliations:** ^1^ Institute of Ophthalmology, University College London, UK

**Keywords:** mitochondria, ageing, fission, fusion, Gerotarget

## Abstract

Mitochondrial function declines with age and is associated with age-related disorders and cell death. In the retina this is critical as photoreceptor energy demands are the greatest in the body and aged cell loss large (~30%). But mitochondria can fuse or divide to accommodate changing demands. We explore ageing mitochondrial dynamics in young (1 month) and old (12 months) mouse retina, investigating changes in mitochondrial fission (Fis1) and fusion (Opa1) proteins, cytochrome C oxidase (COX III), which reflects mitochondrial metabolic status, and heat shock protein 60 (Hsp60) that is a mitochondrial chaperon for protein folding.

Western blots showed each protein declined with age. However, within this, immunostaining revealed increases of around 50% in Fis1 and Opa1 in photoreceptor inner segments (IS). Electron microscope analysis revealed mitochondrial fragmentation with age and marked changes in morphology in IS, consistent with elevated dynamics. COX III declined by approximately 30% in IS, but Hsp60 reductions were around 80% in the outer plexiform layer.

Our results are consistent with declining mitochondrial metabolism. But also with increased photoreceptor mitochondrial dynamics that differ from other retinal regions, perhaps reflecting attempts to maintain function. These changes are the platform for age related photoreceptor loss initiated after 12 months.

## INTRODUCTION

Ageing is driven by multiple mechanisms that give rise to progressive deterioration in cell structure and function leading to cell death. One key theory of ageing relates to mitochondrial function. Mitochondria provide the energy for cellular function in the form of adenosine triphosphate (ATP). However, progressive mutations in mitochondrial DNA (mtDNA) result in reduced ATP production and an increase in reactive oxygen species (ROS) output that is pro-inflammatory [1, 2]. Hence, systemic inflammation becomes a feature of ageing. While we now know that there are many caveats to this theory [3, 4] it retains elements of acceptance.

The photoreceptor population of the outer retina has the greatest energy demand and largest mitochondrial population in the body [5] and this environment is prone to progressive inflammation and cell loss with age [6–8]. This can progress into disease in humans in the form of age-related macular degeneration (AMD) where there is central retinal atrophy. AMD is the leading cause of visual loss among the elderly in the Western world [9–11].

Mitochondria are not static but have the ability to respond to their environment undergoing fission and fusion in response to metabolic or environmental stress, which in turn helps regulate ATP production and minimise the accumulation of mtDNA damage [12]. Fusion helps to reduce stress by mixing the contents of partially damaged mitochondria and is stimulated by energy demand and stress, while fission creates new mitochondria, removes those that are damaged and is also associated with apoptosis [13]. This dynamic network is important in modulating cellular redox status, mtDNA integrity, cellular function and cell death [12]. The balance of these events is disrupted in ageing, which compromises the ability of mitochondria to respond to environmental changes [14, 15].

There is an association between mitochondrial dysfunction and age-related retinal pathophysiology [16] but here attention has largely focused on instability of the mitochondrial genome [17, 18] and mitochondrial dysfunction resulting from oxidative stress [9, 19]. Little attention has been paid to changes that occur during ageing. Here we examine changes in mitochondrial dynamics that are associated with ageing in the mouse retina by examining changes in fission and fusion proteins, (Fis1 and Opa1), heat shock protein 60 (Hsp60), which is predominantly mitochondrial and mitochondrial encoded cytochrome C oxidase subunit 3 (COX III). We assessed mitochondrial dynamics and function in young and old mice to reveal how these change with age in this metabolically demanding environment. Further, in old mice we assessed this in a period prior to the onset of age related photoreceptor loss which occur after one year old [7]. Hence, our data provide a window on the mitochondrial platform that may underpin the process of age related photoreceptor death. Our results show that mitochondrial dynamics decrease with age in the retina but are significantly upregulated specifically in photoreceptor inner segments.

## RESULTS

### Changes in levels and distribution of mitochondrial fission and fusion proteins in the ageing retina

**Figure 1:**
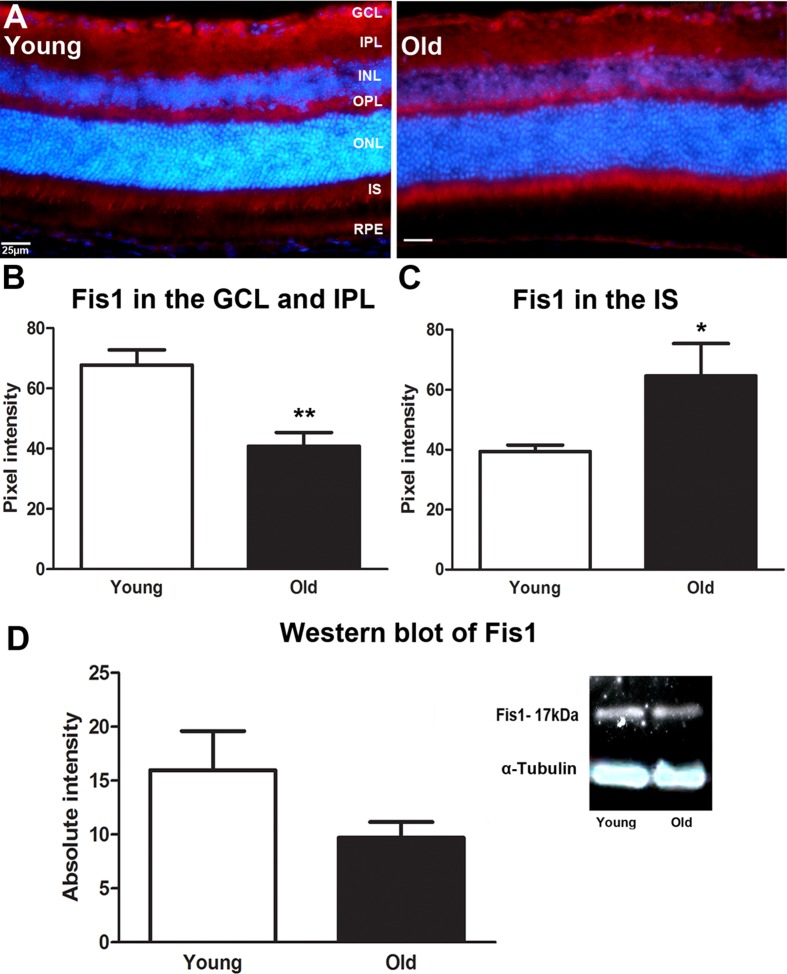
Changes in levels and distribution of mitochondrial fission proteins in the ageing retina of C57BL/6 mice **A.** Retinal sections of both young and old mice were immunostained with a mitochondrial fission antibody, Fis1 (red) and the nuclei counterstained with 4′, 6-diamino-2-phenylindole (DAPI) (Blue). Expression of Fis1 is stronger in the ganglion cell layer of the young retina compared to the old, while the old retina express more Fis1 in the photoreceptor layer than the young. **B.** Graph showing the quantification of Fis1 expression in the ganglion cell layer. There is a significant decrease in Fis1 expression in the ganglion cell layer of the old retina when compared to the young (*P* = 0.0048). **C.** Graph showing that Fis1 is significantly increased in the inner segments of the photoreceptor layer (*P* = 0.0333). **D.** Western blot results for the Fis1 protein showed that Fis1 in the old retina is reduced by 60% when compared to young retinae (*P* = 0.0910). Mean ± SEM. Scale bar = 25μm. GCL; ganglion cell layer, IPL; Inner plexiform layer, INL; inner nuclear layer, OPL; outer plexiform layer, ONL; outer nuclear layer, PR; photoreceptor layer and RPE; retinal pigment epithelial.

We investigate the expression pattern of mitochondrial fission, (Fis1) and fusion, (Opa1) proteins in retinal tissues from young (1M) and old (12M) C57BL/6 mice using immunostaining and Western blot. With immunostaining we quantify differences between young and old where these appear to be obvious. Figure 1A shows that the intensity of Fis1 labelling is significantly stronger in the ganglion cell layer (GCL) and inner plexiform layer (IPL) of the young than the old mouse (*P* = 0.0048, Figure 1B) but it is significantly upregulated in the inner segment of the photoreceptors of the old mouse compare to the young (*P* = 0.0333, Figure 1C). There was no obvious difference in expression with age in the outer plexiform layer (OPL). This suggests a shift in the mitochondrial fission from the ganglion cell layer to photoreceptor inner segments in ageing. Western blot analysis revealed a 60% reduction in the level of Fis1 protein in old mouse even though it was not statistically significant (Figure 1D). However, this was for whole eyecup preparations. Hence, while this protein declined in the whole eye with age it actually increased in the outer retina.

**Figure 2:**
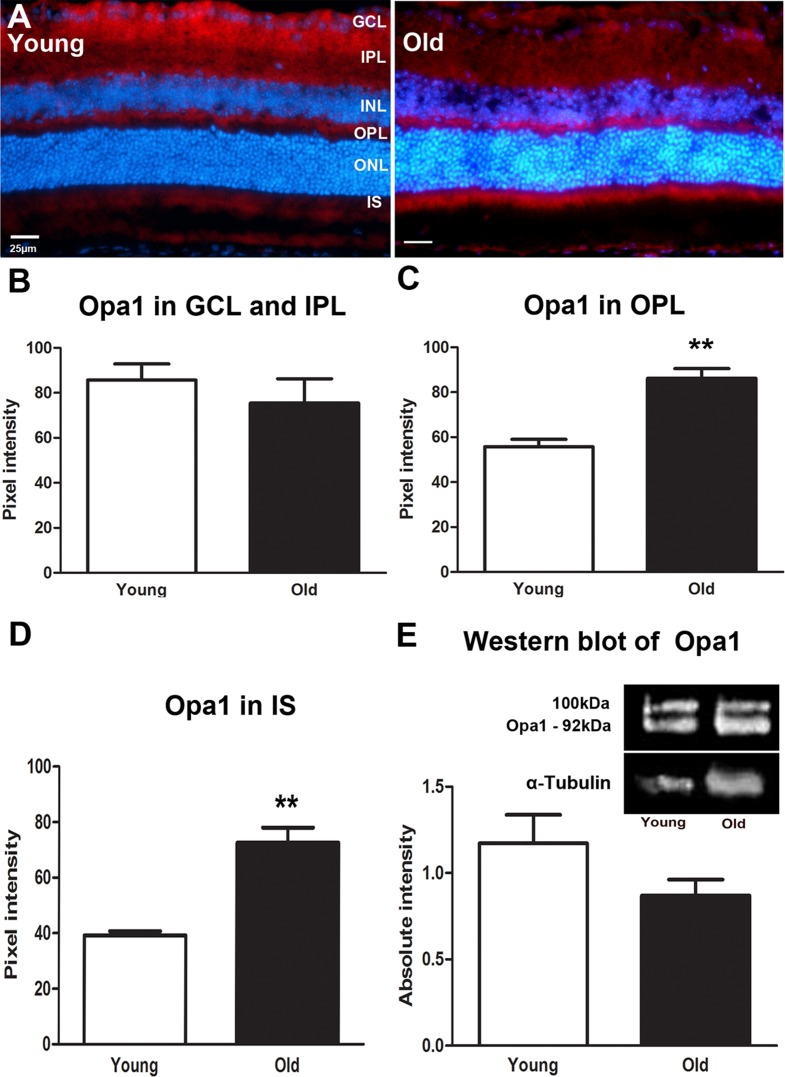
Changes in levels and distribution of mitochondrial fusion proteins in the ageing retina of C57BL/6 mice **A.** Representative micrographs of retinal sections immunostained with a fusion marker, Opa1 (red) and the nuclei counterstained with 4′, 6-diamino-2-phenylindole (DAPI) (Blue). Opa1 is present in the ganglion cell layer and in the outer plexiform layer of young mice while in the old it is present in the outer plexiform layer and the photoreceptor layer. **B.** Opa1 is expressed less in the old than the young retina. **C.** Opa1 expression is significantly higher in the outer plexiform layer of the old retina compared to the young (*P* = 0.0048). **D.** Opa1 expression in the photoreceptor layer. Opa1 is strongly expressed in the photoreceptor layer of the old retina when compared to the young (*P* = 0.0048). **E.** Western blot measurements of Opa1 protein revealing that it is reduced in the old retina compared to young but this did not reach statistical significance (*P* = 0.1276). Mean ± SEM. Scale bar = 25μm. GCL; ganglion cell layer, IPL; Inner plexiform layer, INL; inner nuclear layer, OPL; outer plexiform layer, ONL; outer nuclear layer, PR; photoreceptor layer and RPE; retinal pigment epithelial.

As with Fis1, Opa1 expression was marked in the inner retina in young mice but this pattern shifted with age as shown in Figure 2A. In old mice it is reduced in expression in the IPL and GCL (Figure 2B) but this did not reach statistical difference. With age, Opa1 was significantly higher in expressions in the OPL (*P* = 0.0048, Figure 2C) and in photoreceptor inner segments (*P* = 0.0048, Figure 2D) compare to the young mice. When Opa1 protein was quantified using Western blot there was a 30% reduction in older mice, although this did not reach statistical significance (Figure 2E). Hence, again, this protein declined in the whole eye with age, but increased in the outer retina.

### Mitochondrial metabolic profile and stress

The metabolic profile of mitochondria in response to age was assessed by examining cytochrome C oxidase subunit III (COX III) and Hsp60 expression.

**Figure 3:**
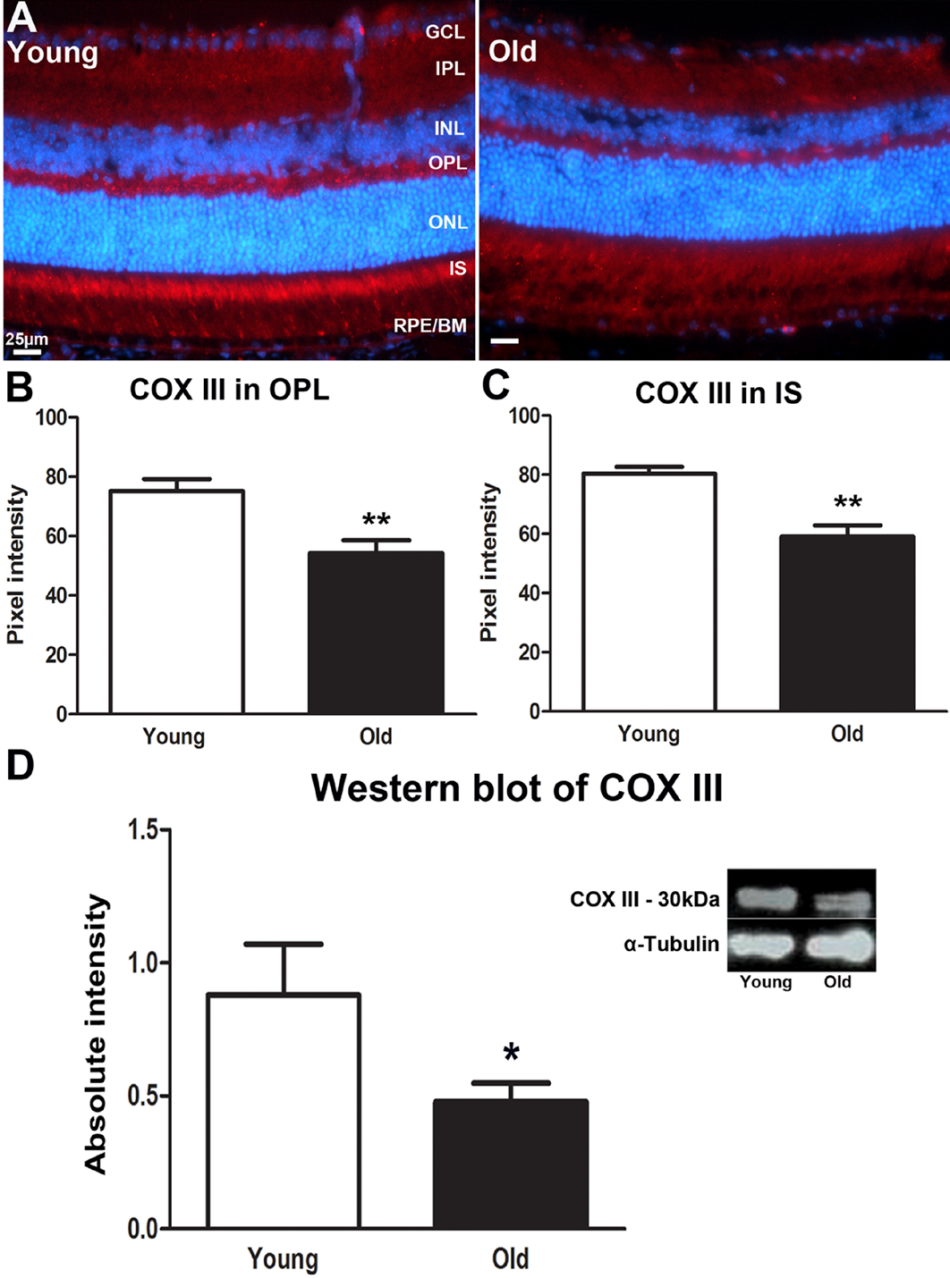
Changes in levels of mitochondrial cytochrome C oxidase III proteins in the ageing retina of C57BL/6 mice **A.** Retinal sections of both young and old mice immunostained for cytochrome C oxidase III (COX III) antibody (red) and nuclei counterstain with 4′, 6-diamino-2-phenylindole (DAPI) (Blue). COX III strongly labelled photoreceptor inner segments in the young retina. **B.** The expression of COX III in the outer plexiform layer is significantly stronger in young mice compared to old ones (*P* = 0.0040). **C.** Measurement of COX III expression in the inner segment of the photoreceptor of young and old mice. There is a significant decrease in the expression of COX III in inner segment of the old retina than in the young retina (P = 0.0040). **D.** Western blot analysis of COX III in young and old mice showing a significant decrease in the retina of old mice compared to young (*P* = 0.0349). Mean ± SEM. Scale bar = 25μm. GCL; ganglion cell layer, IPL; Inner plexiform layer, INL; inner nuclear layer, OPL; outer plexiform layer, ONL; outer nuclear layer, PR; photoreceptor layer and RPE; retinal pigment epithelial.

COX III protein is a mitochondrial-coded subunit on COX protein complex IV that is encoded by the MT-CO3 gene and plays an important role in the regulation of energy transduction in cytochrome oxidase, especially in the assembly and stability of subunits I and II [20]. Reduction of COX III has been associated with apoptosis [21, 22]. In young mice COX III was expressed in the OPL and in photoreceptor inner segments (Figure 3A). In both regions it declined significantly with age (*P* = 0.004 for both, Figure 3B and 3C). Western blot measurement of COX III revealed a similar significant decline in the retina of old mice compare to the young (Figure 3D, *P* = 0.0349). The decreased expression of COX III implies a reduced efficiency of ATP/energy production in the retina of old mice.

**Figure 4:**
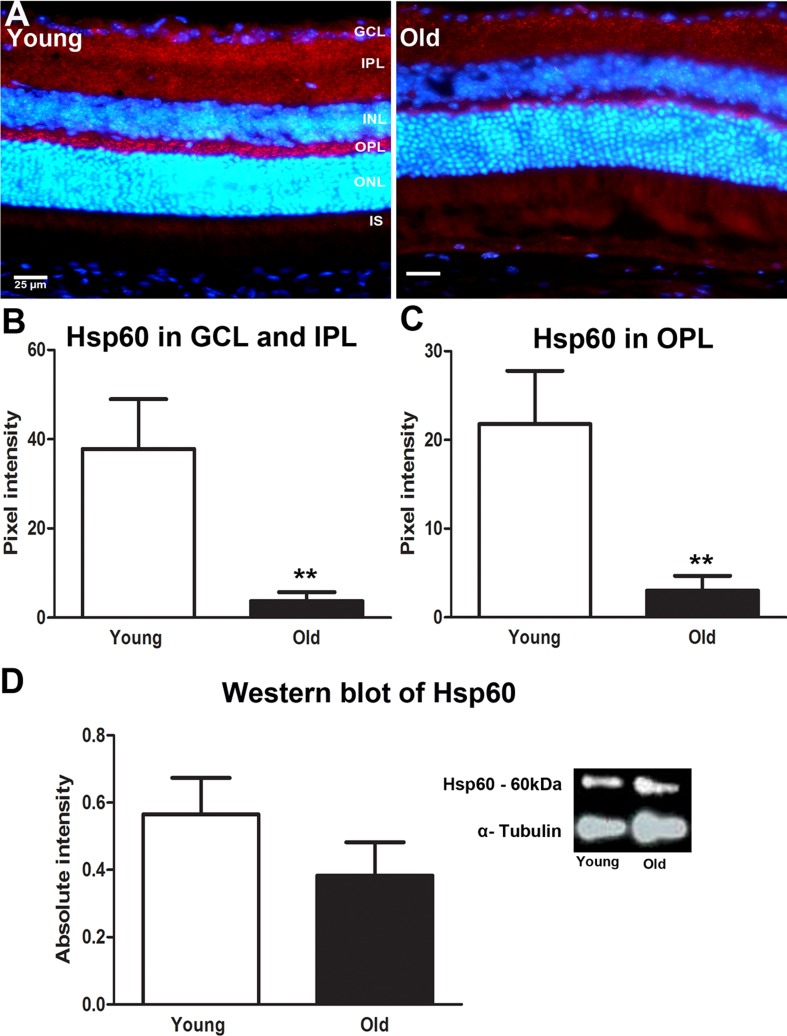
Changes in levels of mitochondrial stress marker Hsp60 in the ageing retina of C57BL/6 mice **A.** Retinal sections of young and old mice immunostained for Hsp60 (red) and the nuclei counterstained with 4′, 6-diamino-2-phenylindole (DAPI) (Blue). Hsp60 is strongly expressed in the inner plexiform layer and outer plexiform layer of the young mice compared to old. **B.** Quantification of the Hsp60 expression in the inner plexiform layer showed that it was significantly reduced in old compared with young (*P* = 0.0043). **C.** Quantification of Hsp60 in the outer plexiform layer showed a significant reduction in the old retina compared to the young (*P* = 0.0022) **D.** Western blot analysis showed a reduction in retinae of old mice which was not statistically significant (*P* = 0.0928). ). Mean ± SEM. Scale bar = 25μm. GCL; ganglion cell layer, IPL; Inner plexiform layer, INL; inner nuclear layer, OPL; outer plexiform layer, ONL; outer nuclear layer, PR; photoreceptor layer and RPE; retinal pigment epithelial.

Hsp60 is a nuclear coded, constitutively expressed heat shock protein resident in the mitochondrial matrix. It is a molecular chaperone with an important role in the handling of damaged mitochondrial [23]. Hsp60 has been shown to both prevent and promote apoptosis [24]. Immunostaining for Hsp60 showed wide distribution across retinal layers (Figure 4A). It was significantly reduced in both the GCL and IPL (*P* = 0.0043) and in the OPL (*P* = 0.0022) with age (Figures 4B and 4C). However, while there was an approximate 30% reduction in Hsp60 with age in Western blots, this was not statistically significant (*P* = 0.0840, Figure 4C). These data suggest that mitochondria in the old retina may be less able to repair oxidative damage that occurs in response to ageing.

### Ultrastructural changes in mitochondria in ageing retina

**Figure 5:**
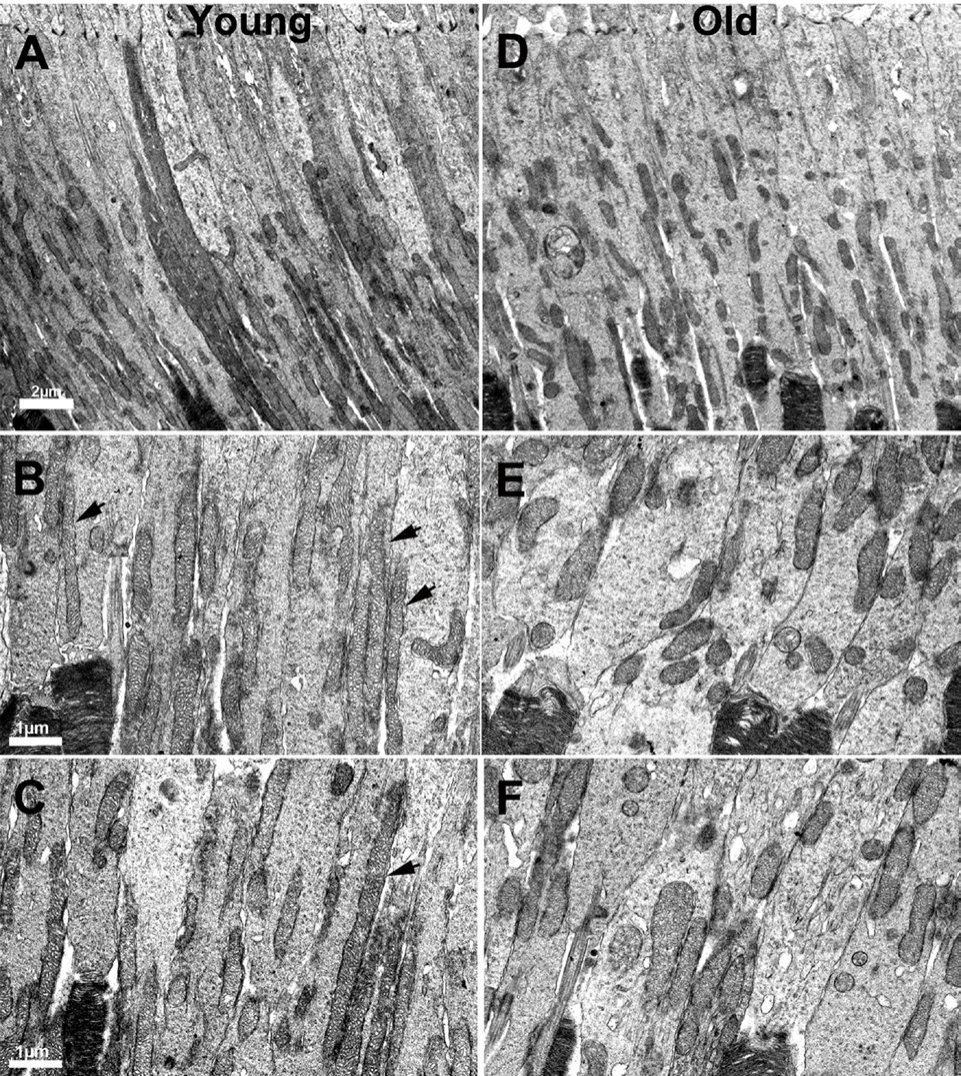
Changes in the morphology of mitochondria in ageing **A.** Low power electron micrograph from young retinae showing elongated and tubular like mitochondria present in photoreceptor inner segments. **B.–C.** Electron micrograph from young retinae at higher magnification showing thin tubular and elongated mitochondria present in the inner segment along the membrane (black arrows). **D.** Electron micrograph of low magnification from an old mouse showing mitochondria that are relatively fragmented, being shorter in photoreceptor inner segments. **E.–F.** Electron micrograph from old mice showing fragmented mitochondria that are shorter and fragmented compared to those found in young animals.

**Figure 6:**
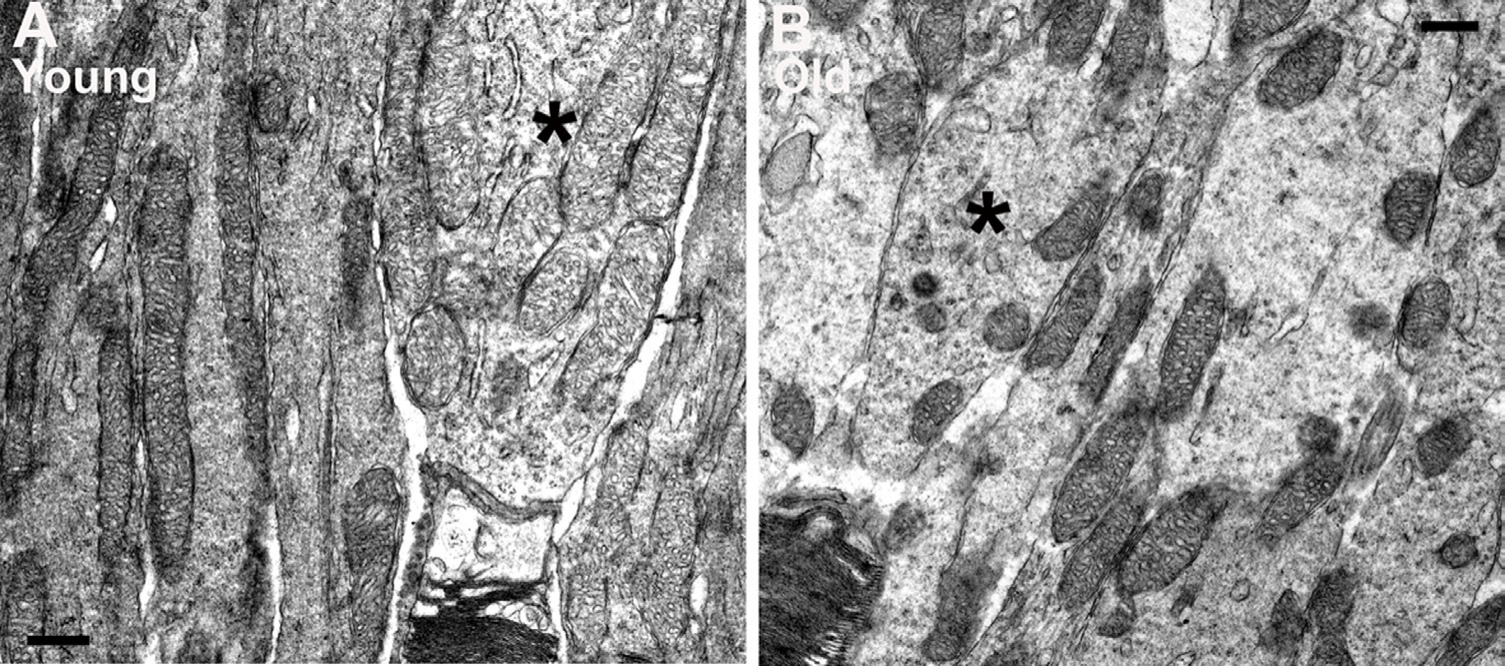
Differences in mitochondria morphology between cones and rods in young and old C57BL/6 mice **A.** Representative electron micrograph of young inner segment showing mitochondria in rod and cone photoreceptors. The mitochondria in rods are condensed and electron dense while the mitochondria in the cones are relatively electron translucent. **B.** Representative electron micrograph from an old mouse at the level of the inner segment. There are fewer mitochondria in the cone of old mice and these are electron dense similar to those found in adjacent rods. Scale bar = 0.5 μm.

Mitochondria were clearly identified in all of the electron microscopic images from each animal in photoreceptor inner segments. There were clear differences with age. In low power images from young mice (Figure 5A–5C) mitochondria were mainly elongated and aligned along the inner segment membrane. In old mice they were relatively fragmented, being much shorter and relatively swollen and a greater proportion were found located away from the cell membrane (Figure 5D–5E). There were clear differences between rods and cones, with cones having a higher mitochondrial density and also their mitochondria being less electron dense, consistent with a higher intracellular ATP concentration [25]. Because of the larger cone inner segment and their high mitochondrial density, a greater proportion of mitochondria in young mice were located away from the cone cell membrane. In spite of this, those not against the membrane were often abutted directly to those that were. However, with age cone mitochondrial density dropped and they became more electron dense resembling those seen in rods, which may reflect reduced ATP within them (asterisk (*) in Figure 6).

**Figure 7:**
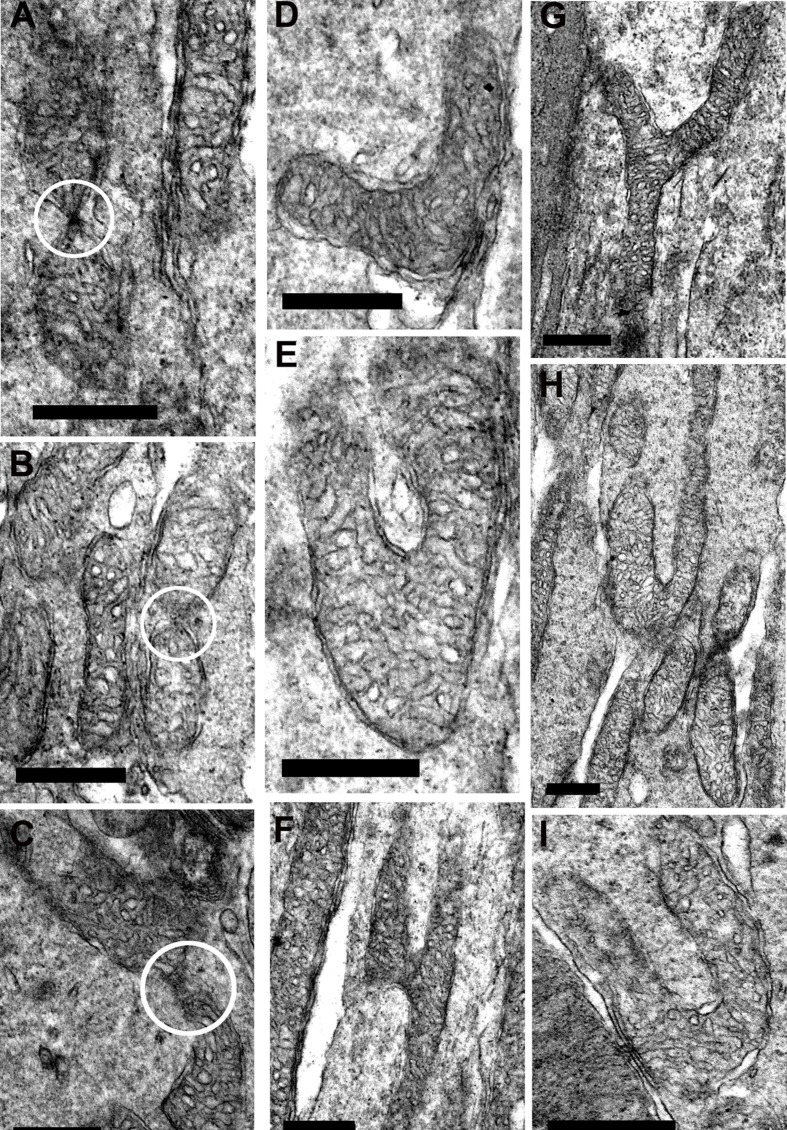
Fission and fusion in inner segment of young mice **A.–C.** are electron micrographs of mitochondria undergoing either fission or fusion (white circle) in the inner segment. **D.–I.** Representative electron micrographs of suspected mitochondrial fusion based on the criteria of Bereiter-Hahn and Voth [25] who showed using real time imaging that the branching in E and F resulted in fusion. The bends seen in **D.**, **E. H.** and **I.** are associated with greater motility. Scale bar = 0.5 μm.

**Figure 8:**
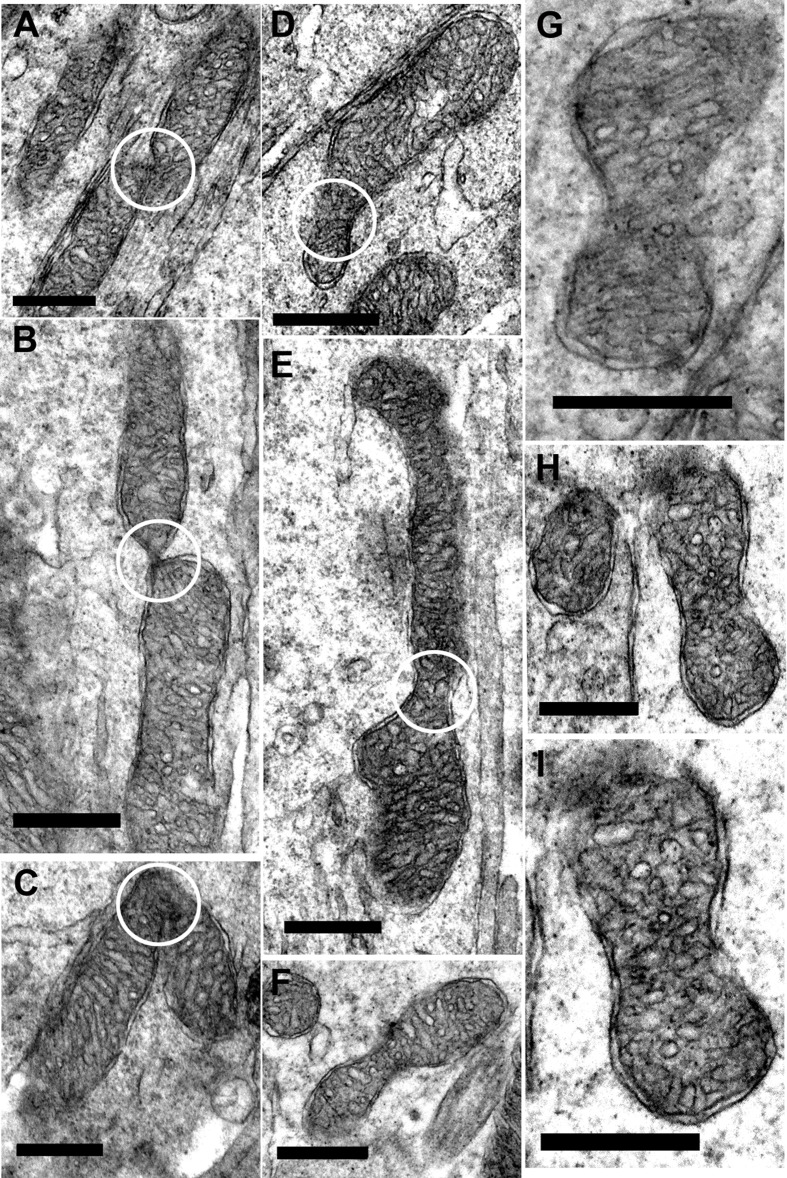
Fission and fusion in inner segment of old mice **A.–C.** Representative electron micrographs of mitochondria undergoing fission/fusion in the inner segment of old mice (white circle) where the mitochondrion is either dividing or fusing. **D.–I.** Electron micrographs of suspected mitochondrial fusion/dividing in the old mice. (White circle indicate the ‘pinching off’ of the mitochondria for either fission or fusion). Scale bar = 0.5 μm.

At higher power in young mice, there was evidence of both fusion and fission, although such profiles were not common. Here fusion and fission were classified as locations where mitochondria appear pinched as if they were separating or joining as shown in Figure 7A–7C. However, a marked feature was the numerous bends or turns in the long mitochondria that in real time imaging have been associated with mitochondrial motility [25](Figure 7D and 7E, 7H and 7I). There were also extensive branches (Figure 7F–7G). In real time imaging of mitochondrial dynamics, these morphological features have been shown to be the result of mitochondrial fusion [25]. Direct morphological comparison between young and old are problematic, as the number of mitochondria between these two groups is likely to be different, hence the samples may not be the same. However, a striking feature of mitochondria in old retinae was the high incidents of morphological features that could be linked to fusion or fission. As in younger animals, mitochondria appeared to be pinched as if separating or joining (Figure 8A–8E). However, there were also many mitochondria that had morphologies that appeared to be linked to fission with a pinched dumbbell appearance (Figure 8F–8I) that were rarely seen in young animals.

## DISCUSSION

Our results show that fission and fusion proteins decline with age in the retina, consistent with other studies [14, 15, 26, 27] who showed that mitochondrial dynamics significantly decrease with age. However, this decrease does not occur in photoreceptors as here both proteins are significantly up regulated when they were examined in sections, implying that at this location mitochondrial dynamics actually increased with age. The reason for this may be linked to two key factors. First, the metabolic demand of the outer retina is very high, so this region is more prone to age related stress. Second, the vascular supply to the outer retina comes from the choroidal vessels that sit on the other side of Bruch’s membrane. Bruch’s membrane changes significantly with age. Between 2 and 8 months it thickens significantly by approximately 40% [28] and over this period there is a >30% decline in retinal ATP [29]. As our aged animals were 12 months old, it is likely that thickening of Bruch’s membrane was greater than 40%. Such thickening will be associated with reduced metabolic exchange that will restrict the oxygen supply to photoreceptors and their oxygen demanding mitochondria. Further, the choroidal circulation is not responsive to the metabolic state of photoreceptors, which means that the outer retina can suffer from chronic hyperoxia or hypoxia [30, 31]. Hence, with age the outer retina experiences increased stress that may be related to subsequent patterns of photoreceptor loss [7, 8]. This increased stress may drive the response seen in photoreceptor mitochondria revealed here.

The differences in mitochondrial morphology in photoreceptor inner segments with age were striking. It has been reported previously that ageing mitochondria tend to fragment, consistent with what we have shown [15]. Both fusion and fission were upregulated in photoreceptors with age when tissue was immunostained, but the EM images revealed additional patterns. The mitochondrial branching commonly seen in young animals has been shown to be a feature of fusion, which is viewed as protective [25, 32]. Such patterns were rare in old mice. However, as they were associated with elongated mitochondria that were absent in old animals a direct comparison is difficult to make and it does not mean that this process was absent. Rather, it is possible that the morphological platform on which it occurs has changed. Likewise the curved profiles of many elongated mitochondria in young mice may simply have arisen because it was the only way to accommodate their length although it has also been associated with dynamic behavior [25].

As elongated mitochondria with their branching patterns were a feature of youth, so the large incidents of dumbbell profiles in fragmented mitochondria were a key feature in photoreceptor inner segments in old mice. After examining many such profiles we tentatively view them as potential features of fission, but have no direct evidence for this. Consequently, there are multiple interpretations of the different EM images seen in the two groups. However, again while there are marked differences in mitochondrial morphology with age, interpretations must be conservative.

Alterations in mitochondrial dynamics modulate their biogenic activity [33–36]. Therefore the balance between fission and fusion is likely to be key in retinal health and changes in this balance are strongly linked to disease [32]. There is emerging recognition that disruptions to mitochondrial dynamics contribute widely to disease, and this is not restricted to those that are classically recognized as purely mitochondrial in nature [32]. In light of this, it has recently been shown in a murine model of age related macular degeneration based on the complement factor h knock out mouse, that ATP production declines significantly prior to the development of the retinal phenotype [37, 38].

Age related upregulation of mitochondrial dynamics in photoreceptor inner segments is associated with a significant reduction of COX III. COX III is encoded by the mitochondrial genome and is one of the components of the COX catalytic core [39]. COX drives electrons that flows from cytochrome c to molecular oxygen and promotes the proton pump used to produce ATP. Therefore reductions in COX efficiency are related to declining ATP production and elevated reactive oxygen species (ROS), resulting in cellular damage [40–42]. The decrease in COX III expression shown here implies a reduced efficiency of electron transport, oxidative phosphorylation and ATP/energy production in retina of aged mice [3, 27]. Such ageing-induced alterations in mitochondrial activities are likely to contribute to increased mitochondrial oxidative stress and mtDNA mutations in the aged retina [3, 29].

Ageing is associated with a decline in cell stress responses [43, 44]. Hsp60 is a highly conserved chaperon that assists mitochondrial protein folding and facilitates proteolytic degradation of denatured proteins in an ATP-dependent manner [45]. It is normally up regulated in response to both acute and chronic stress [46, 47]. Reductions in Hsp60 expression may precipitate apoptosis [48]. We show significant down regulation of Hsp60 with age. As Hsp60 requires ATP, the decline shown here in ageing is expected [45]. Consequently, the aged retina cannot mount an appropriate stress response at the mitochondrial level to counter the impact of ageing.

This study reveals numerous significant changes in mitochondrial morphology and associated protein expression in the ageing retina at 12 months, a period by which retinal ATP production has declined by around 40% [29]. However, the retina at this age shows few if any significant structural changes. Li et al. [49] used the same mice as in this study but failed to find any significant differences in photoreceptor numbers between 2 and 12 months of age. Likewise, Mustafi et al. [50] found none between 1 and 8 months of age in their control group. As the mouse retina is approximately 97% rod dominated [51] this must predominantly reflect stability of rod numbers, although Cunea et al. [8] have presented evidence for cone loss at 12 months restricted to medium/long wavelength sensitive cells in the periphery. Evidence for rod loss beyond 12 months is widely present [52]. In spite of this, at 12 months there is clear loss of both rod and cone photoreceptor function as reflected in attenuated amplitudes in the electroretinogram [49, 53], but not in their implicit timing [49] or their rate of adaptation [53]. Interestingly, Mustafi et al. [50] show reductions in rod and cone ERG amplitudes at 8 months, but these were not statistically significant. Taken together with our data, these results support the notion that the changes we document in mitochondria may undermine photoreceptor function as represented specifically by the amplitude of rod and cone responses. However, such changes in mitochondrial function at this stage are not sufficient to reduce photoreceptor numbers.

Ageing is associated with not only increased damage, but also an impaired ability to repair. Our results support the notion that retinal ageing is associated with impaired balance in mitochondrial dynamics and highlight the importance of mitochondria in ageing and cellular integrity in the retina.

## MATERIALS AND METHODS

### Animals

1 and 12 month old C57BL/6 mice were used in this study (N = 25 per group). Animals were housed under a 12 hour light /12 hour dark cycle with access to food and water ad libitum. All animals were used with University College London ethics committee approval conformed to the United Kingdom Animal License (Scientific Procedures) Act 1986 (UK). UK Home Office project license (PPL 70/6571).

### Immunohistochemistry

Mice were culled by cervical dislocation. The eyes from both groups of mice (N = 5 per group) were collected and processed for cryosection. The enucleated eyes were fixed in buffered 4% paraformaldehyde pH 7.4 for 1h and cryopreserved in 30% sucrose in phosphate buffered saline (PBS) and then embedded in optimum cutting temperature (OCT) compound (Agar Scientific Ltd). 10μm cryosections were thaw-mounted on a slide and incubated for 1h at room temperature in a 5% Normal Donkey serum in 0.3% (v/v) Triton X-100 in PBS, pH 7.4. They were then incubated overnight with either a rabbit polyclonal antibody to Fis1 (1:200, Abcam), a rabbit polyclonal antibody to Opa1 (1:300, Abcam), a rabbit polyclonal antibody to heat shock protein 60 (Hsp60) or a goat polyclonal antibody to cytochrome c oxidase subunit 3 (COX III) (1:100, Santa Cruz Biotechnology, Inc.) diluted in 1% Normal Donkey Serum in 0.3% Triton X-100 in PBS. After primary antibody incubation, sections were washed several times in 0.1M PBS and incubated in their respective secondary antibody, donkey anti-rabbit conjugated with Alexa Fluor 568 (Invitrogen) or donkey anti-goat conjugated with Alexa Fluor 568 (Invitrogen) made up in 2 % Normal Donkey Serum in 0.3% Triton X-100 in PBS at a dilution of 1:2000 for 1 hour at room temperature. Negative controls were undertaken by omitting the primary antibody. After the secondary antibody incubation, sections were washed several times and nuclei were subsequently stained with 4′, 6-diamidino-2-phenylindole (DAPI) (Sigma) for 1 min. Slides were then washed in 0.1 M PBS followed by several washes in Tris buffered Saline (pH 7.5). Slides were mounted in Vectashield (VECTOR Laboratories) and coverslipped. Fluorescence images were taken in JPEG format at X400 using an Epi-fluorescence bright-field microscope (Olympus BX50F4, Japan).

### Western Blot

Eyes (*N* = 20 for each group) were dissected on ice and the retina and RPE-choroidal tissues were collected and snap frozen in liquid nitrogen. Samples were homogenised in 2% SDS with protease inhibitor cocktail (Sigma-Aldrich, UK), and centrifuged at 13,000 X g. The supernatant was transferred to a new microcentrifuge tube and protein concentration was measured with an absorbance of 595nm and Bovine Serum Albumin was used as a standard protein concentration.

Equal amounts of proteins were separated by a 10% sodium dodecyl sulfate-polyacrylamide gel electrophoresis and electrophoretically transferred onto nitrocellulose membranes. The nitrocellulose membranes were pre-treated with 5% non-fat dried milk in 1M PBS (pH7.4) for 2 hrs and incubated overnight with either a rabbit polyclonal antibody to Fis1 (1:500, Abcam), a rabbit polyclonal antibody to Opa1 (1:1000, Abcam), a rabbit polyclonal antibody to heat shock protein 60 (Hsp60) (1:20000, Abcam) or a goat polyclonal antibody to cytochrome c oxidase subunit 3 (COX III) (1:500, Santa Cruz Biotechnology, Inc.) followed by several washes in 0.05% Tween-20 in 1M PBS. The membranes were then incubated with either a goat anti rabbit IgG peroxidase conjugated secondary antibody (1:2000, Thermo Scientific) or a rabbit anti goat IgG peroxidase conjugated secondary antibody (1: 2000) for 1h. Immunoreactivity was visualised by exposing x-ray film to blots incubated with ECL reagent (SuperSignal West Dura, Thermo Scientific). Total protein profile was determined by staining membrane with 2% Ponceau S solution to check that the extraction and transfer of proteins were consistent. Protein bands were then photographed and scanned. The absolute intensity of each band was then measured using Adobe Photoshop CS5 extended.

### Electron microscope morphological analysis

Animals (N=3 per group) were sacrificed and both eyes were immersed in Karnovsky’s fixative (3 % glutaraldehyde, 1% paraformaldehyde in 0.07M sodium cacodylate) for 2 hours at room temperature. The eyes were dissected and the anterior chamber and lens were removed and they were then dehydrated and embedded in Epon. Ultrathin sections were cut and then stained with lead citrate and viewed on a JEOL 1010 transmission electron microscope and images captured using Gatan Orius SC1000B charge-coupled device camera

### Analysis

#### Measurement of the expression of Fis1, Opa1, Hsp60 and COX III in the retina by immunostaining

Fluorescence images were taken in JPEG format at X400 using an Epi-fluorescence bright-field microscope and pixel intensity was measured using Adobe Photoshop CS5 extended. The lasso tool was used to draw a line around the areas of interest.

#### Measurement of the Fis1, Opa1, Hsp60 and COX III in the retina and RPE using Western blot

Scanned images of the protein gel were inverted to grayscale format and the mean gray value was measured for each protein band by using the lasso tool to draw a line all the way around the edges of the band using Adobe Photoshop CS5 extended. The absolute intensity was calculated by multiplying the mean gray value and the pixel value.

### Statistical analysis

A Mann-Whitney U test was used to compare the two groups. Data was analysed using Graph pad Prism, version 5.0 (Graphpad, San Diego, CA).
